# Impact of birth tourism on health care systems in Calgary, Alberta

**DOI:** 10.1186/s12913-022-07522-4

**Published:** 2022-01-28

**Authors:** Simrit Brar, Mruganka Kale, Colin Birch, Fiona Mattatall, Medini Vaze

**Affiliations:** 1 Department of Obstetrics and Gynecology, University of Calgary, Calgary, Canada; 2 Department of Family Medicine, University of Calgary, Calgary, Canada

**Keywords:** Birth tourism, Medically uninsured

## Abstract

**Background:**

Birth tourism refers to non-resident women giving birth in a country outside of their own in order to obtain citizenship and/or healthcare for their newborns. We undertook a study to determine the extent of birth tourism in Calgary, the characteristics and rationale of this population, and the financial impact on the healthcare system.

**Methods:**

A retrospective analysis of 102 women identified through a Central Triage system as birth tourists who delivered in Calgary between July 2019 and November 2020 was performed. Primary outcome measures were mode of delivery, length of hospital stay, complications or readmissions within 6 weeks for mother or baby, and NICU stay for baby.

**Results:**

Birth Tourists were most commonly from Nigeria (24.5%). 77% of Birth Tourists stated that their primary reason to deliver their baby in Canada was for newborn Canadian citizenship. The average time from arrival in Calgary to the EDD was 87 days. Nine babies required stay in the neonatal intensive care unit (NICU) and 3 required admission to a non NICU hospital ward in first 6 weeks of life, including 2 sets of twins. The overall amount owed to Alberta Health Services for hospital fees for this time period is approximately $694 000.00.

**Conclusion:**

Birth Tourists remain a complex and poorly studied group. The process of Central Triage did help support providers in standardizing process and documentation while ensuring that communication was consistent. These findings provide preliminary data to guide targeted public health and policy interventions for this population.

**Supplementary Information:**

The online version contains supplementary material available at 10.1186/s12913-022-07522-4.

## Definitions

***Birth Tourism: ***the practice of non-residents of a country traveling to a new country with the intention to give birth in the new country.

***Birth Tourist***: an Uninsured Prenatal Patient who is a non-resident who travels to a new country with the intention of giving birth there. In Canada, Birth Tourists do not qualify for publicly funded health care coverage, even if they are Canadian citizens, because they do not reside in Canada. Occasionally Birth Tourists have private health insurance that partially or entirely covers their medical bills.

***Uninsured Prenatal Patient:*** a patient who does not have provincial healthcare but who resides in Canada. They may not have Alberta Healthcare Coverage (AHC) for various other reasons, including.

- ***Convention Refugee***: a person who meets the refugee definition in the 1951 Geneva Convention relating to the Status of Refugees (UN Refugee Agency, 1951). This definition is used in Canadian law and is widely accepted internationally. *Health Care costs for Convention Refugees are covered by the federal government.*
- ***Refugee Claimant or Asylum Seeker: ***a person seeking refugee status whose case has not been decided.
- ***Other:*** A pregnant patient who is neither a Convention Refugee nor Birth Tourist, but resides in Alberta. These patients may have an expired Canadian Work Permit, Visitor Visa, or Student Visa. They may also be undocumented meaning residing in Canada without legal documentation.

***Jus soli: ***the principle of ***jus soli*** allows for a child born on Canadian soil to a visiting foreign national to obtain Canadian citizenship (Canadian Citizenship Act, RSC 1985, c.C-29).

## Background

The term Birth Tourism refers to the practice of a country traveling to a new country to give birth there for a variety of personal reasons. Such reasons may include: to obtain Citizenship for the infant in that country (*jus soli)*; the notion that the costs of medical care are lower in that country compared to the individual’s home country; the perception that medical care in that country is safer; and potential access to public schooling, healthcare, and sponsorship of other family members in the future. In recent years, discussions regarding Birth Tourism have been more prominent globally [1]. This has been a relevant topic in the United States, Canada, and in Hong Kong. It is difficult to capture the true extent of Birth Tourism. There is no legal requirement to capture this information nor is this information a prerequisite for a birth certificate [2].

A 2018 Canadian study showed an increase in both the proportion and absolute numbers of babies born to foreign nationals in nearly all provinces [3]. However, hospital coding used to capture uninsured patients does not selectively capture Birth Tourists. There are many other circumstances that may result in a nonresident service code including those described in our definitions under the ‘Uninsured Prenatal Patient’ [3]. Anecdotal reports indicate rising concern about the excess burden placed on the Canadian public healthcare system; many Canadian provinces are facing significant cost overruns within the health care system resulting in limited capacity to provide obstetrical care to local residents [4]. However, there has never been any large scale data collection regarding the actual numbers of Birth Tourists. There has also not been data collection regarding the payment of bills for service provided by those identified as Birth Tourists [5].

There are many unique aspects in the care provision of Birth Tourists and Uninsured Prenatal patients. For many providers in Canada, the notion of private payment for medical care is foreign and uncomfortable. Providers may be unsure of how to undertake the conversation with consistency and may not have the time required to do so in a systematic way. Additionally, many pregnant immigrant women without medical insurance often receive inadequate prenatal care [6]. As Jarvis et al. (2019) reviewed, this population is more likely to present late in pregnancy for care, receive less prenatal testing, and to receive inadequate prenatal follow up. Poor prenatal care has been associated with poor birth outcomes including increased risk of preterm delivery and low birth weight [6]. This is further compounded by the differences in medicolegal protection for providers when treating uninsured patients. The Canadian Medical Protective Association’s Governing Law and Jurisdiction Agreement was created to assist in ‘establishing Canadian jurisdiction for any potential legal actions that may result from care or treatment provided by Canadian physicians or healthcare organizations to non-residents’ [7] [8]. The complexity of all of these issues can be very difficult for care providers to navigate.

Increasing Birth Tourism in Calgary, Alberta, has raised similar concerns about costs to the healthcare system and the access to care for both Uninsured Prenatal patients and Birth Tourists. There was a lack of standardized process to identify and appropriately coordinate medical care for this population. The Uninsured Prenatal patient population is often more complex with multiple social issues. In our opinion, this was a far more complex group to initially establish a consistent streamlined process for. As such, the Department of Obstetrics and Gynecology worked closely with Low Risk Provider groups within Calgary to establish a consistent and streamlined process for Birth Tourists arriving in Calgary. This process would require distinguishing Birth Tourists from other patients without provincial health insurance. Data Integration, Measurement and Reporting (DIMR), which is current standard of data extraction, does not separate birth tourists from non-birth tourists. Subsequently, we hoped these changes would help to better support Uninsured Prenatal patients. However, that process has not been streamlined to the same degree at this time.

Our primary objective was to collect detailed information on pregnant persons without Alberta Health Care (AHC) coverage in order to identify Birth Tourists in Calgary, evaluate their clinical outcomes, and characterize their financial and resource burden on the healthcare system. Our secondary objective was to collate the information collected to contribute to advancing the literature on Birth Tourism as this topic is relevant across the globe.

## Procedures for patients without AHC

We developed a centralized intake process for prenatal patients without AHC delivering in Calgary. Starting in July 2019, all prenatal referrals in Calgary for patients without AHC coverage were redirected to a Central Triage (CT) office. This office was consistently run by one administrator with the support of a three member physician team. Patients were administered a questionnaire and subsequently triaged to a care provider based on patient preference, risk profile and city quadrant of residence. Care providers, with the exception of midwifery who had a separate process for billing of patients, then invoiced CT directly. Invoices were required to be consistent with what would have been billed to Alberta Health and were reviewed by the physician leads for the same e.g. excess modifier codes could not be billed. In addition to ensuring standardized payments for services, CT ensured that the Canadian Medical Protective Association’s Governing Law and Jurisdiction form for all providers and Alberta Health Services was reviewed and completed as well as consent for information sharing among providers as well as Alberta Health (AH). Patients stayed in contact with CT with respect to when they would be leaving the country and timing of refund. In situations where English was not the primary language, language line or other translation services (through family or physician) was used. Refunds were issued once all invoices were received (approximately eight weeks postpartum). If a patient refused CT, we endeavored to follow up with how and where the patient received care.

A ‘Central Triage package’ was reviewed and provided to all patients deemed to be Birth Tourists in person, via email, or via registered mail. Within this package, there was full disclosure of the concern regarding potential cost burden of Birth Tourism within Calgary and the rationale for the creation of CT – particularly to deter the process of Birth Tourism. A deposit of $15,000 was collected and held in trust by CT from each Birth Tourist to cover cost of physician service fees. In alignment with both the Alberta Medical Association (AMA) and the College of Physicians and Surgeons of Alberta (CPSA), patients were also able to pay for their fees individually following the service and care was never refused. The physician service fees for Birth Tourists were billed at a rate of five times the rate determined by AH in accordance with the Uninsured Services guidance document provided by the Alberta Medical Association (AMA) [8]. Service fees for uninsured patients were not above standard Schedule of Medical Benefits (SOMB) rates and deposits ranged from no deposit to $2500. A $300 administrative fee was invoiced to Birth Tourists to pay for administrative services. The physician administrators did not receive any payment for their administrative role. Information regarding the SOMB codes, AMA and CPSA was provided to all of the patients.

The CT system also sought to create a system by which uninsured patients could be identified quickly and thus be provided support letters and advocacy for more timely access to AHC given the impending medical care required. These scenarios were also assessed on a case by case basis allowing for provider discretion. Scenarios involving Uninsured Prenatal patients were found to be more complex with more individuation required which was another reason to not formally include them in the analysis. Our past experience with Uninsured Prenatal patients showed often face significant economic barriers that would make it difficult to pay an advance deposit for medical services. These patients were referred to an appropriate obstetrical provider with full understanding that appropriate medical care would be provided regardless of ability to pay. Physician fees for services provided to Convention Refugees were billed directly to the federal government.

Although a deposit was collected by CT to cover the cost of physician services, a deposit was not collected to cover Alberta Health Services (AHS) site fees for hospital stay which are different for each hospital. AHS site fees are charged based on a daily rate. As part of the CT package, all Birth Tourists were given a copy of the current AHS site fee schedule and were made aware that the AHS finance office would bill them directly at the time of hospital admission.

## Methods

The questionnaire was developed by the study authors (see Appendix 1). It included open ended questions exploring the patient’s reasons for pursuing childbirth in Canada, and specifically in Calgary. We collected information regarding the patient’s country of origin (we utilized the patient’s stated Country or place of current residence), citizenship, date and port of entry to Canada, refugee status, and the type of visa possessed by the patient. For patients who lived in Alberta prior to pregnancy, we asked questions regarding how long they had lived in Alberta/Canada, employment history in Alberta/Canada and whether they previously held AHC insurance or provincially funded health care insurance from another Canadian province or territory. The questionnaire did not screen for specific medical conditions, but we did ask patients whether they had any pre-existing medical conditions or history that would potentially impact delivery. This better allowed CT to triage to the appropriate care provider.

The CT team identified Birth Tourists by questionnaire responses in the following ways: (1) patient self-identifying as a Birth Tourist (2) the Birth Tourist arrived in Canada while pregnant, on a visitor visa, and had no intention to live permanently in Canada immediately following the delivery. Women who were classified as Uninsured Prenatal Patients were excluded. If there was a case that was unclear, the case was brought to the three-member physician team and reviewed sometimes with the support of hospital social work. There were no cases where consensus was not achieved through this method. Most patients were administered the questionnaire during the antenatal period, although, some presented for the first time at the hospital during the time of delivery. In this circumstance, the delivering physician would refer the patient to CT and attempts were made to administer the questionnaire postpartum. Occasionally a deposit was also collected post- partum to streamline invoice payment.

Physicians sent invoices for services provided directly to CT, who then paid physicians from the deposit. An itemized receipt for all physician services was provided to each Birth Tourist and a refund was issued to the Birth Tourist where applicable. Any billing codes used were done so in the standard of the Alberta SOMB and were required to match the service provided. The physician invoices including the billing codes were also used to determine mode of delivery or any additional care required. Comments provided on these invoices or from the patients following delivery to administration at CT were also collected.

We completed a qualitative descriptive analysis using information obtained from the CT patient questionnaires and physician invoices from July 1, 2019 to November 1, 2020. Only questionnaires completed by persons identified to be Birth Tourists were included in the review, with all identifying information removed. A data dictionary was used to collect the variables analyzed (Appendix 2). Data on delivery and readmission for Birth Tourists was obtained through provider invoices as well as patient reporting.

A cost analysis of physician service fees and AHS hospital site fees was performed by review of AHS hospital stay financial data as well as review of the physician invoices provided.

A waiver of consent was obtained for this study. Most of the Birth Tourists included in the analysis were no longer in Calgary during the study period and the resources required to contact all patients exceeded the resources available to the study team. No patients or care providers were directly contacted. The study team reviewed existing data, documents and records excluding the hospital chart. Ethics approval for this study was obtained from the Conjoint Health Research Ethics Board at the University of Calgary (REB20-0026). This study was unfunded.

## Results

In total, 102/227 patients captured by CT from July 15, 2019 to November 1, 2020 were identified as Birth Tourists. The 125 patients who were not Birth Tourists but instead Uninsured Prenatal patients were not included in the analysis. 89/102 (87%) patients were identified by direct referral to CT. The remaining 14 (14%) Birth Tourists were identified after delivery by invoices from physicians. The average and median maternal age was 32 years (range 20–45, SD 5.5). Of these women, 42% had not had a previous live birth, 42% were multiparous, and parity of 16% was unknown. There were 5/102 (5%) Birth Tourists who self-referred to midwives for home birth or birth center delivery. 83/102 (81%) had an encounter with an AHS Calgary zone hospital. Of the remaining 19 patients (19%), 8 were lost to follow up, 2 delivered elsewhere in Alberta, 3 went back to their home country for delivery (Mexico, Trinidad, and Tanzania, respectively), 3 stated they would seek Midwifery care for home birth within Calgary, and 3 went to Ontario. There were 17 (17%) Birth Tourists that did not have a Regional Health Number.

83% of patients stated they came to Canada with a Visitor Visa. The type of visa for 14% of patients was unknown, 2% had possessed a Student Visa, and 1 patient was a Canadian Citizen who had never lived in Canada. The date of arrival in Canada was known for 77/102 patients; the average time from arrival in Calgary to the expected due date was 87 days (SD 76) with a range of 7 to 502 days. Exact date of departure from Calgary is known for 34/102 patients; for these patients, the average length of stay in Calgary after delivery was 49 (range 18–80) days;

Birth Tourists were most commonly from Nigeria (25%), Middle East (18%) China (11%), and India (8%) and Mexico (6%). There were no birth tourists from Western Europe or Australia. 77% of Birth Tourists stated that their primary reason to deliver their baby in Canada was for the desire for a Canadian baby who would be eligible for Canadian citizenship. 8% stated their reason to deliver in Canada was to access better health care. 40% of questionnaire respondents chose Calgary specifically because they had family and/or friends in the city. We were unable to expand further beyond this on other reasons for delivery in Canada or outside their home countries.

38% of patients delivered vaginally and 35% delivered by cesarean Sect. 29% of deliveries were of unknown type. Two mothers required readmission to hospital within 6 weeks postpartum; one was admitted to ICU for 5 days for cardiac reasons, and another was admitted for severe preeclampsia and stroke. Another patient had severe postpartum preeclampsia but discharged herself against medical advice. Her subsequent outcome is not known. Nine babies required stay in the neonatal intensive care unit (NICU) and 3 required admission to a non NICU hospital ward in first 6 weeks of life, including 2 sets of twins. The average length of stay was 18 days with a range of 1–63 days.

With respect to maternal health history prior to arrival in Canada: 9 had a history of previous cesarean section, 6 patients reported having some form of Diabetes Mellitus, 3 arrived with a cerclage in place, 2 arrived acutely hypertensive, 2 had history of myomectomy, 2 had threatened preterm labour (one actively in preterm labour), 2 had blood borne infection, and 2 had known twin pregnancies. There was one new diagnosis of HIV following arrival in Canada.

There was AHS billing information captured for 83/102 (81%) Birth Tourists (Appendix 2). The average amount of fully paid AHS maternal invoices was $6234.92 (Appendix 2). The average amount of fully paid AHS neonatal invoices was $4185.91 (Appendix 2). There were 8 cases (10%) where AHC was received for the newborn but there was an outstanding invoice for the mother (Appendix 2). There were 17 cases (21%) where there was an unpaid neonatal bill and 29 cases with an unpaid maternal bill (35%) (Appendix 2). The outstanding fee amount is known for 29 mothers and 17 newborns (including 2 sets of twins) (Table 1, 2 and 3). As of the date of this report, approximately $290,000.00 of fees remain outstanding for the 29 mothers (average $9704.62; range $948- $72,445), and approximately $404,000.00 remains outstanding for 17 newborns (average $23, 747.65) (Table 1, 2 and 3).

**Table 1:** Birth Tourists with Partially Paid AHS invoices with Up-to-Date Payment Plan

Maternal AHS Invoice Paid($)	Outstanding Maternal Invoice($)	Neonatal AHS Invoice Paid($)	Outstanding Neonatal AHS Invoice ($)	Referral to CT	CT deposit paid	Central Triage Deposit Refund Amount ($)
23,942.00	20,557.25	5149.00	80,881.00	Yes	Yes	19,766.03
11,088.00	13,217.00	6062.00	45,880.00	Yes	No	Nil

**Table 2:** Birth Tourists with Partially Paid AHS invoices with Outstanding Payment Plan

Maternal AHS Invoice Paid($)	Outstanding Maternal Invoice($)	Neonatal AHS Invoice Paid($)	Outstanding Neonatal AHS Invoice ($)	Referral to CT	CT deposit paid	Central Triage Deposit Refund Amount ($)
12,041.50	41,421.00	14,232.00	119,735.50	Yes	Yes	27,568.79
5614.00	18,030.85	Nil	96,064.50	Yes	No	Nil
2954.50	5648.00	Nil	2150.00	No	No	Nil

**Table 3:** Birth Tourists with Unpaid AHS invoices with No Payments

Maternal AHS Invoice Paid($)	Outstanding Maternal Invoice($)	Neonatal AHS Invoice Paid($)	Outstanding Neonatal AHS Invoice ($)	Referral to CT	CT deposit paid	Central Triage Deposit Refund Amount ($)
Nil	23,989.50	Nil	Unknown^a^	Yes	Yes	11,801.61
Nil	18,030.85	Nil	Unknown^a^	Yes	No	Nil
Nil	21,485.00	Nil	Nil	Yes	No	Nil
Nil	121,837.25	Nil	34,367.50	No	No	Nil

^a^Unknown as many are covered by AHC

Of the Birth Tourists identified through CT, 54/88 (61%) paid the $15 000 deposit for physician services (Appendix 2). Of these, 7 had additional invoices above the total deposit amount (range $300- $2760.92). Two of those individuals did not pay the outstanding invoice ($980 and $2219.29). For the majority of these patients (89%), the 15,000.00 deposit was adequate for physician fees, and the patient received a refund (avg. $5484.70). Of the Birth Tourists identified through CT, 34/88 (39%) did not pay the deposit. Reasons provided included: 10 patients indicated that they paid the provider (obstetrician, family physician, or midwife) directly for fees incurred, 20 refused to pay with no other reason provided, one self-referred to midwifery for delivery at home, no reason was provided for the remaining 3 individuals who did not pay the CT deposit.

## Discussion

Of the 227 patients captured by CT, 125 (55%) were deemed to not be Birth Tourists and were classified as Uninsured Prenatal patients. As stated earlier, this group was not included in the analysis. However, we do feel it prudent to state that we feel a process is urgently required to support this population. Since the initiation of this project, the CT team has been able to directly liaise with AHS to help expedite AHC insurance for those that qualify. We would advocate for a similar process that is available to all eligible women across the province. Having a system in place to distinguish Uninsured Prenatal patients is of benefit to the patient and the provider. It provides a means to better identify and support those for whom health care coverage can be optimized and also allows an opportunity to better support the needs of the patient. The additional support required can be difficult for individual community providers to provide in isolation. Our process did not specifically have a role for social work and additional community supports. However, we do feel it would be beneficial to create a dedicated process to support Uninsured Prenatal patients not only in the acquisition of AHC but also the opportunity for the provision of community and social supports that may be required. Women with an undocumented status may delay prenatal care due to concerns of being deported, and as a result increase adverse outcomes. We feel this population would benefit from additional community support and discussion of ensuring safe treatment without the threat of deportation and also creation of a system where cost is not a barrier to accessing care.

Birth Tourists represented 44% of patients seen by CT. Of these, 87% of Birth Tourists were identified by direct referral to CT. There may be a few explanations for non-referral to CT. There is a complex network of care providers in Calgary which likely led to slow dissemination of the information regarding CT. There was some hesitancy from obstetrical providers to refer to CT perhaps due to pre-existing relationships or the feeling of obligation within communities with higher rates of Birth Tourism, the concern that there would not be a distinction between Birth Tourists and other Uninsured Prenatal patients, and the concern that patients may avoid care altogether. Through standardized communication we were able to address a common issue identified by providers: the fact that Birth Tourists were receiving incorrect and often varied messaging regarding costs associated with delivery, not only from their friends and online forums, but also from different obstetric providers in Calgary. Previously, the practice of different providers charging different fees for the same service led to ‘deal making’ between providers and Birth Tourists. Clear communication and collection of a standardized deposit in advance for physician services mitigated some of this problem. Many potential patients contacted the CT office from overseas prior to their arrival in Canada. Physicians did not have to collect payment in hospital or postpartum, which is often very difficult. It is concerning that 39% of patients did not pay the deposit. The reasons for this remain unclear and were not explored in the breadth of this study. Though some of these patients did ultimately pay for the physician service provided following the delivery, it was not clear to CT if all providers were paid and what amount. 

83/102 (81%) of Birth Tourists had an encounter with an AHS Calgary Zone hospital. Of the remaining 19 patients (19%), 8 were lost to follow up. That is not insignificant and speaks to the need for greater administrative support to ensure that both mother and baby had access to care in a timely fashion. 5% of Birth Tourists identified that they would be self-referring to midwifery specifically for home birth or birth center delivery. Unfortunately, we were not able to capture the financial data with respect to cost of home birth versus birth center in such scenarios. Midwifery care is unique funding model in that there is a direct contract between AHS and the Alberta Association of Midwifery [9]. There are only a certain number of allocated ‘spots’ for patients as such there is a wait list for this model of care. It is possible that the number of patients who transferred to midwifery was in fact higher than 5% as Alberta does not have coordinated data for maternity care that includes home births. This may also provide some explanation for the 17 patients with no Regional Health Number. In addition to potentially delivering at home, they could also have delivered outside of the city or province or may have been an error in invoice creation in that it simply wasn’t added. Due to the difficulty in consistent recording, we were only able to obtain information that one midwifery home birth required a subsequent NICU admission. An additional limitation noted in through our process with the separate process of fee collection and assessment by Midwifery. We feel it would be of benefit to all providers in patients to have one streamlined process for all providers as this avoids duplication and improves efficiency.

The majority 76/102 (75%) of visas granted to patients seen through CT were Visitor Visas. Some patients did state they had taken out private insurance. Our process did not have consistent collection of private insurance information or the extent of coverage. This is a limitation of our study and examination of this in further studies may help to better understand the financial options available to patients.

The average time from arrival in Calgary to delivery/EDD was 87 days with a range of 7–502 days (average 87 days). The 502 days reflects an individual who ultimately left and returned to their home country. This is a limitation of our data in that we are not able to capture exactly when all individuals left. This also underscores the difficulty in capturing a subset population of uninsured and undocumented pregnant women. When the time of stay surpasses 365 days, the individual can no longer be considered as a tourist [10]. The individual who stayed 502 days self-identified as a tourist. We do feel that the data on the uninsured undocumented pregnant women is lacking and further investigation is also required in this area.

The average of 87 days arrival in advance of EDD or delivery demonstrates potentially significant implications in care provision and supports the evidence that many women in this population seek care late in pregnancy. Women who arrive very close to the end of pregnancy or postdates may be in a rush to obtain care. Potential for complications including elevation in maternal blood pressure and stillbirth rise as women approach and pass their due date. This rush for care puts an increased stress of immediate care provision in an already stressed system. Improved education with VISA issuance would be helpful to inform women of the risks they may be taking in traveling to deliver overseas late in pregnancy.

Almost a third of women presenting to CT had a known preexisting medical condition. This included one person actively experiencing preterm labour and three with a cerclage in the current pregnancy. Many Birth Tourists were concerned about hospital stay > 24 h due to the additional daily AHS fee. In some cases this lead to Birth Tourists discharging themselves Against Medical Advice. The potential for increased morbidity is significant. A limitation to our study is that we were not able to assess if advance knowledge of the CT process may have changed the decision to undertake Birth Tourism.

In comparison to the 35% of Birth Tourists delivered cesarean section, the general cesarean section rate in Alberta in 2018 was 31% [11]. The NICU admission rate in Calgary is approximately 7%; whereas the NICU admission rate among neonates born to the Birth Tourist group was 9%.

The time from delivery to departure was 49 days with a range of 18–80 days. Approximately one third of patients did not specify a date of departure but stated they would be leaving ‘after delivery. Though the data is limited by the fact that 1/3 did not specify an actual date, the average of 49 days to departure is consistent with the definition of a Birth Tourist not permanently staying Canada at that given time. This does have implications for the Canadian born child’s ability to access Alberta Health care. Specifically, a person must make Alberta his/her home and be physically present in Alberta at least 183 days in a 12-month period (not including a tourist, transient or visitor). 17% of children born to mothers identified as ‘birth tourists’ received AHC with a third of those mothers still having unpaid bills for maternal care. This poses the question as to whether or not the criteria for obtaining Alberta Health Care for neonates born to mothers without Canadian Health Care is rigorous enough.

The most common reason stated by the patients in our study for travel to Canada to give birth for ‘a Canadian baby’. A Canadian citizenship is perceived as a valuable item not only in the short term but also long term for health care and education. The long term implications to this are complex and multifaceted and should not be simplified. However, our data does show that the Canadian Citizenship is held in value and is provided as reason for Birth Tourism.

The overall amount owed to Alberta Health Services during the study period is almost $700,000 (Table 4). There were two sets of twins during the study period both sets requiring NICU stays. The average length of stay for babies taken to the NICU was 18 days with a range of 1–63 days. One set of twins occupied a NICU bed for 50 and 63 days respectively. NICU bed capacity has been an ongoing concern in Calgary zone with twins contributing to the concern given the need for two beds. The $700,000 amount also did not capture the cost to the system for midwifery care or patients who subsequently left Calgary to deliver in a surrounding area. We are concerned that Birth Tourists may be leaving Calgary to find cheaper care in overburdened surrounding regions. This emphasizes the need for a standard provincial process to provide clear and transparent guidance to both patients and providers. A significant limitation of our study was the inability to account for all possible financial and resources burdens e.g. both outpatient and inpatient imaging and laboratory services, physicians who are funded by Alternate Funding Plans (AFPs), or support from ancillary staff.

**Table 4:** Birth Tourists with Unpaid AHS invoices with No Payments

Total Outstanding Maternal AHS Invoice ($)	247,216.80
Total Outstanding Neonatal AHS Invoice ($)	379,078.50
Total Outstanding AHS Invoices ($)	626,295.30

Our AHS financial data was not clearly outlined in terms of what costs were paid. We do feel that moving forward a clearly itemized invoice for hospital services should be provided to patients as well as CT. There were a number of people who were issued refunds from CT for physician service fees yet still had outstanding AHS fees owing. We propose to only issue CT refunds once a fully paid hospital invoice is shown. A further limitation of our financial analysis was that we did not include foreign parents hiring Canadian surrogates to carry a child who will leave the country.

Another limitation of our study was that all out of country patients were not identified through the CT. These cases were often recognized through communication from ancillary care providers such as pediatrics asking for clarification on billing. Some out of country patients presented for the first time at delivery. There were also some out of country patients making individual arrangements with maternity care providers that also came to light when additional care providers asked for clarification regarding billing. Given the lack of a robust system of data collection, it is very possible that Birth Tourists and Uninsured Prenatal patients were missed. Unfortunately, we were also unable to assess which women remained in Canada after expiration of their visitor visa. There were many scenarios in the financial data were there were outstanding maternal charges, AHC was obtained for the infant and there was no referral to CT. It is possible that some of these women were not truly Birth Tourists but rather Uninsured Prenatal patients that overstayed a visa and we were not able to capture the specifics of their scenario.

Our analysis overlapped with the Covid-19 pandemic. We had anticipated birth tourism to drop to almost zero with the lack of air travel. However, that was not the case, suggesting that at a time of reduced international travel, birth tourism continued. It would be important to collect data on an ongoing basis as we suspect that rates of birth tourism in past and future years may be higher.

## Conclusion

The process of Central Triage did help support providers in standardizing process and documentation while ensuring that communication was consistent.

The process 1) enabled us to differentiate Birth Tourists from non-Birth Tourists, which is something that DIMR data has been previously unable to, and thus identify populations that may need additional support 2) ensure that physicians are more likely to get paid for services rendered 3) standardize a fee schedule across the city for Birth Tourists. We do feel that an area of further investigation that is required is better understanding the non-Birth Tourist population. This group seems to have greater complexity in terms of background and potential need for support.

With increasing strain on healthcare budgets, outstanding patient invoices warrant attention. In a publicly funded system that is often under strain, we do feel that our process of CT did help to improve efficiency and provide more timely access to appropriate care for Birth Tourists. We do believe that having a clearly delineated policy for Birth Tourists would allow for better collection of data, consistency of messaging and communication between all parties involved in health care from the patient to the frontline worker to the administrator. There may be unintended consequences of such policy on the non-Birth Tourist group, in particular the undocumented patient. It would be imperative that the determination of Birth Tourists was very clear and not reliant on self –identification alone.

## Supplementary Information

**Additional file 1. **

**Additional file 2: **

**Additional file 3. **

## Data Availability

The datasets used and/or analyzed during the current study are available from the corresponding author on reasonable request.
