# Supplementary material for: Impact of birth tourism on health care systems in Calgary, Alberta
Source: BMC Health Serv Res. 2022 Jan 28;22:120. doi: 10.1186/s12913-022-07522-4 (PMC8798307; doi:10.1186/s12913-022-07522-4)
Supplement: Supplementary file 2 — Additional file 2: [file 12913_2022_7522_MOESM2_ESM.docx]

**Data Dictionary**

1. Outstanding bill Maternal
2. Outstanding bill newborn
3. AHS Bill Comments
4. Age at presentation
5. Country of Origin
6. Port of Entry
7. EDD
8. Date arrived in Canada
9. Difference between EDD and Date arrived in Canada
10. Type of visa
11. Why delivery in Canada
12. Why delivery in Calgary
13. Parity
14. Date left Canada
15. Date of Delivery
16. Type of Delivery
    1. Vaginal delivery
    2. Caesarean Section
17. Maternal health conditions
18. Additional maternal hospital care within 6 wks.
    1. Yes
    2. No
19. Additional newborn hospital care within 6 weeks
    1. Yes
    2. No
20. Referred to Central Triage
    1. Yes
    2. No
21. Central Triage Deposit paid
    1. Yes
    2. No
22. Central Triage refund processed
    1. Yes (amount)
    2. No
23. Additional payment invoiced by Central Triage
    1. Yes (amount
    2. No
24. Additional invoice paid
    1. Yes
    2. No
25. Comments
