# Supplementary material for: Impact of birth tourism on health care systems in Calgary, Alberta
Source: BMC Health Serv Res. 2022 Jan 28;22:120. doi: 10.1186/s12913-022-07522-4 (PMC8798307; doi:10.1186/s12913-022-07522-4)
Supplement: Supplementary file 3 — Additional file 3. [file 12913_2022_7522_MOESM3_ESM.docx]

**Appendix 2: Supplementary Tables**

**Supplementary Table 1: Characteristics of Birth Tourists with Paid in Full AHS invoices and Referral to CT with CT Deposit Paid**

|  | **Total Maternal AHS Invoice ($)** | **Total Neonatal AHS Invoice ($)** | **Country of Origin** | **Central Triage Deposit Refund Amount ($)** |
| --- | --- | --- | --- | --- |
| 1 | 12,557.50 | 35,125.00 | China | Nil |
| 2 | 6,777.50 | AHC | China | 2760.92 |
| 3 | 5,830.00 | 2,117.50 | China | 3405.15 |
| 4 | 13,455.00 | 2,117.50 | China | 263.68 |
| 5 | 17,490.00 | 4,235.00 | China | Nil |
| 6 | 5,717.50 | 3,047.50 | China | 2393.90 |
| 7 | 5,825.00 | 2,117.50 | China | 6799.15 |
| 8 | 5,830.00 | 2,117.50 | Cyprus | 4730.28 |
| 9 | 4,520.00 | 2,150.00 | Dubai | 7148.20 |
| 10 | 4,982.50 | 3,047.50 | El Salvador | 5855.35 |
| 11 | 10,762.50 | 2,117.50 | Egypt | 200.50 |
| 12 | 5,830.00 | 3,912.50 | Ghana | 89.40 |
| 13 | 4,085.00 | AHC | Ghana | 6318.15 |
| 14 | 4,927.50 | AHC | Greece | 2394.80 |
| 15 | 7,625.00 | 7,045.00 | Hong Kong | 576.45 |
| 16 | 4,927.50 | 3,015.00 | India | 8576.90 |
| 17 | 10,752.50 | 2,117.50 | India | 4666.95 |
| 18 | 4,982.50 | 2,150.00 | India | Nil |
| 19 | 9,856.00 | AHC | Jordan | 7518.50 |
| 20 | 5,415.00 | 2,117.50 | Jordan | 2219.29 |
| 21 | 8,957.50 | 2,117.50 | Jordan | 8035.60 |
| 22 | 10,537.50 | 2,150.00 | Kuwait | 4519.55 |
| 23 | 11,660.00 | 4,235.00 | Kuwait | 4202.85 |
| 24 | 1,993.00 | 860.00 | Lebanon | Nil |
| 25 | 11,660.00 | 4,235.00 | Nigeria | 300.00 |
| 26 | 5,830.00 | 2,117.50 | Nigeria | 6174.00 |
| 27 | 4,030.00 | 2,117.50 | Nigeria | 6422.85 |
| 28 | 3,620.00 | 2,117.50 | Nigeria | 7422.10 |
| 29 | 4982.50 | 2150.00 | Nigeria | 7303.15 |
| 30 | 19,783.13 | 785,197.50 | Nigeria | 635.25 |
| 31 | 4932.50 | Unknown | Nigeria | 7760.50 |
| 32 | 5,880.00 | 2,150.00 | Nigeria | 9520.50 |
| 33 | 8,142.50 | 4,300.00 | Nigeria | 5512.80 |
| 34 | 5717..50 | 2,150.00 | Pakistan | 8862.40 |
| 35 | 4,820.00 | 2,150.00 | Philippines | 6147.30 |
| 36 | 4,085.00 | 2,150.00 | Republic of Guinea | 7415.75 |
| 37 | 7,620.00 | 2,117.50 | South Sudan | 4407.80 |
| 38 | 6,012.00 | 21,828.50 | Sudan | 175.80 |
| 39 | 17,815.50 | 2,150.00 | Unknown | 8032.45 |
| 40 | Unknown | Unknown | Vietnam | 8120.81 |

**Supplementary Table 2: Characteristics of Birth Tourists with Paid in Full AHS invoices and No Referral to CT with No CT Deposit Paid**

|  | **Total Maternal AHS Invoice ($)** | **Total Neonatal AHS Invoice ($)** | **Country of Origin** |
| --- | --- | --- | --- |
| 1 | 897.50 | Unknown | Mexico |
| 2 | 4985.50 | 2150.0 | Saudi Arabia |
| 3 | 4927.50 | 2,117.50 | United Arab Emirates |

**Supplementary Table 3: Characteristics of Birth Tourists with Paid in Full AHS invoices and Referral to CT with No CT Deposit Paid**

|  | **Total Maternal AHS Invoice ($)** | **Total Neonatal AHS Invoice ($)** | **Country of Origin** |
| --- | --- | --- | --- |
| 1 | 5,415.00 | 2,117.50 | Egypt |
| 2 | 1,390.00 | AHC | El Salvador |
| 3 | 2,807.75 | AHC | Ethiopia |
| 4 | 4,927.50 | 2,117.50 | Iran |
| 5 | 4,927.50 | 2,117.50 | Ivory Coast |
| 6 | 4,517.50 | 2,117.50 | Lebanon |
| 7 | 897.50 | Unknown | Nigeria |
| 8 | 4,927.50 | 2,117.50 | Nigeria |

**Supplementary Table 4: Characteristics of Birth Tourists with Partially Paid AHS invoices with Up to Date Payment Plan and Referral to CT with CT Deposit Paid**

| **Maternal AHS Invoice Paid($)** | **Outstanding Maternal Invoice ($)** | **Neonatal AHS Invoice Paid($)** | **Outstanding Neonatal Invoice ($)** | **Country of Origin** | **CT Deposit Refund Amount ($)** |
| --- | --- | --- | --- | --- | --- |
| 6,727.00 | 371.25 | 2,640.00 | 47,045.00 | China | 6930.50 |
| 10,762.50 | Nil | 511.00 | 31,599.00 | Pakistan | 875.46 |
| 5,786.50 | 5,157.00 | AHC | Nil | Philippines | 3077.02 |
| 666.00 | 15,029.00 | 1,998.00 | 2,237.00 | Taiwan | 8883.05  3. |

**Supplementary Table 5: Characteristics of Birth Tourists with Partially Paid AHS invoices with Up to Date Payment Plan and No CT Deposit Paid**

| **Maternal AHS Invoice Paid($)** | **Outstanding Maternal Invoice($)** | **Neonatal AHS Invoice Paid($)** | **Outstanding Neonatal Invoice($)** | **Country of Origin** | **Refer to CT** |
| --- | --- | --- | --- | --- | --- |
| 4,030.00 | Nil | 6,062.00 | 45,880.00 | Nigeria | Yes |
| 1,271.50 | 8,060.00 | AHC | Nil | Mexico | No |
| 5,786.50 | 5,157.00 | AHC | Nil | Mexico | No |

**Supplementary Table 6: Characteristics of Birth Tourists with Partially Paid AHS invoices with Outstanding Payment Plan or Collections and Referral to CT with CT Deposit Paid**

| **Maternal AHS Invoice Paid($)** | **Outstanding Maternal Invoice ($)** | **Neonatal AHS Invoice Paid($)** | **Outstanding Neonatal Invoice ($)** | **Country of Origin** | **CT Deposit Refund Amount ($)** |
| --- | --- | --- | --- | --- | --- |
| Nil | 11,660.00 | 4,235.00 | Nil | China | 5632.75 |
| 3,572.00 | 948.00 | Nil | 3,047.50 | Columbia | 6345.00 |
| 423.00 | 5,889.50 | 423.00 | 1,694.50 | Egypt | 1440.10 |
| 4,000.00 | 30.00 | 3,015.00 | Nil | Ghana | 6368.94 |
| 1,681.50 | 3733.50. | Nil | 2,117.50 | Iran | 1778.40 |
| Nil | 11,660.00 | 6,559.00 | 105,826.00  $105,826.00  $105,826.00  $105,826.00  $105,826.00 | Nigeria | 4568.95 |
| 2,365.00 | 7,500.00 | Nil | 7,050.00 | Nigeria | 1434.65 |

**Supplementary Table 7: Characteristics of Birth Tourists with Partially Paid AHS invoices with Outstanding Payment Plan or Collections and No CT Deposit Paid**

| **Maternal AHS Invoice Paid($)** | **Outstanding Maternal Invoice($)** | **Neonatal AHS Invoice Paid($)** | **Outstanding Neonatal Invoice($)** | **Country of Origin** | **Refer to CT** |
| --- | --- | --- | --- | --- | --- |
| 830.00 | 9,922.50 | AHC | Nil | India | Yes |
| 4,784.00 | 6,076.00 | Nil | 96,064.50 | Nigeria | Yes |
| Nil | 2,032.85 | AHC | Nil | Sudan | Yes |
| 5,114.50 | Nil | 4,116.08 | 24,631.50 | Nigeria | No |
| 897.50 | 4,085.00 | Nil | 2,150.00 | Pakistan | No |
| 2,057.00 | 1,563.00 | AHC | Nil | Unknown | No |

**Supplementary Table 8: Characteristics of Birth Tourists with Unpaid AHS invoices with No Payments and Referral to CT with CT Deposit Paid**

| **Maternal AHS Invoice Paid($)** | **Outstanding Maternal Invoice ($)** | **Neonatal AHS Invoice Paid($)** | **Outstanding Neonatal Invoice ($)** | **Country of Origin** | **CT Deposit Refund Amount ($)** |
| --- | --- | --- | --- | --- | --- |
| Nil | 6499.5 | AHC | Nil | China | 1673.70 |
| Nil | 5,830.00 | Unknown | Nil | Ethiopia | 4426.51 |
| Nil | 11,660.00 | Unknown | Nil | Nigeria | 5701.40 |

**Supplementary Table 9: Characteristics of Birth Tourists with Unpaid AHS invoices with No Payments and No CT Deposit Paid**

| **Maternal AHS Invoice Paid($)** | **Outstanding Maternal Invoice($)** | **Neonatal AHS Invoice Paid($)** | **Outstanding Neonatal Invoice($)** | **Country of Origin** | **Refer to CT** |
| --- | --- | --- | --- | --- | --- |
| Nil | 10,937.50 | AHC | Nil | Mexico | Yes |
| Nil | 5620.00 | AHC | Nil | Nigeria | Yes |
| Nil | 4,927.50 | Unknown | Nil | Iran | Yes |
| Nil | 4517.50. | Nil | 2,117.50 | Central America | No |
| Nil | 72,445.00 | Nil | 32,250.00 | Ghana | No |
| Nil | 6,670.00 | Unknown | Nil | India | No |
| Nil | 10,752.25 | Unknown | Nil | Unknown | No |
| Nil | 27,452.50 | Nil | Nil | USA | No |
